# Maternal obesity and baseline vitamin D insufficiency alter the response to vitamin D supplementation: a double-blind, randomized trial in pregnant women

**DOI:** 10.1093/ajcn/nqab112

**Published:** 2021-05-08

**Authors:** Raghad M Alhomaid, Maria S Mulhern, Jj Strain, Eamon Laird, Martin Healy, Michael J Parker, Mary T McCann

**Affiliations:** Nutrition Innovation Centre for Food and Health (NICHE), School of Biomedical Sciences, Ulster University, Coleraine, Northern Ireland; Department of Food Sciences and Human Nutrition, College of Agriculture and Veterinary Medicine, Qassim University, Buraydah, Saudi Arabia; Nutrition Innovation Centre for Food and Health (NICHE), School of Biomedical Sciences, Ulster University, Coleraine, Northern Ireland; Nutrition Innovation Centre for Food and Health (NICHE), School of Biomedical Sciences, Ulster University, Coleraine, Northern Ireland; School of Biochemistry and Immunology, Trinity College Dublin, Dublin, Republic of Ireland; Department of Biochemistry, Central Pathology Laboratory, St. James's Hospital, Dublin, Republic of Ireland; Department of Obstetrics and Gynaecology, Western Health and Social Care Trust, Altnagelvin Area Hospital, Londonderry, Northern Ireland; Nutrition Innovation Centre for Food and Health (NICHE), School of Biomedical Sciences, Ulster University, Coleraine, Northern Ireland

**Keywords:** maternal obesity, body weight, BMI, pregnancy, vitamin D, 25(OH)D concentration, vitamin D requirements

## Abstract

**Background:**

The achievement of target 25-hydroxyvitamin D [25(OH)D] concentrations in pregnancy may be altered by maternal obesity.

**Objective:**

The authors examined the effects of maternal supplementation of 10 μg compared with 20 μg vitamin D_3_/d on maternal and umbilical cord 25(OH)D. The secondary aim was to investigate the influence of maternal BMI (in kg/m^2^) on the response of the primary outcomes.

**Methods:**

The authors performed a 2-arm parallel double-blind randomized trial with 240 pregnant women recruited throughout the year in Northern Ireland. Women were stratified by BMI to receive 10 or 20 µg vitamin D_3_/d from 12 gestational wk (GW) until delivery. Maternal blood samples collected at 12, 28, and 36 GW and from the umbilical cord were analyzed for total serum 25(OH)D. A total of 166 women completed the study.

**Results:**

Mean ± SD 25(OH)D at 36 GW was 80.8 ± 28.2 compared with 94.4 ± 33.2 nmol/L (*P* < 0.001) (10 compared with 20 µg vitamin D_3_/d, respectively). In those classified with 25(OH)D <50 nmol/L at baseline and assigned 10 µg vitamin D_3_/d, mean 25(OH)D concentrations remained <50 nmol/L at 36 GW, whereas those <50 nmol/L at baseline and assigned 20 µg vitamin D_3_/d, had mean 25(OH)D concentrations ≥50 nmol/L at 28 and 36 GW. In women with obesity and 25(OH)D <50 nmol/L at baseline, the related mean umbilical cord 25(OH)D was deficient (<25 nmol/L) in both treatment groups, whereas those with obesity and 25(OH)D ≥50 nmol/L at baseline had an average umbilical cord 25(OH)D between 25 and 50 nmol/L in both treatment groups.

**Conclusions:**

Supplementation of 20 µg vitamin D_3_/d is needed to attain maternal and umbilical cord 25(OH)D concentrations ≥50 nmol/L on average, in those who start pregnancy with low 25(OH)D concentrations (<50 nmol/L). Under current recommendations, women with obesity and low 25(OH)D in early pregnancy are particularly vulnerable to maintaining a low 25(OH)D concentration throughout pregnancy and having an infant born with deficient 25(OH)D concentrations. This trial was registered at ClinicalTrials.gov as NCT02713009.

## Introduction

Obesity in pregnancy is a risk factor for low concentrations of maternal and neonatal vitamin D, commonly referred to as 25-hydroxyvitamin D [25(OH)D] (1). Vitamin D metabolism is altered during pregnancy to meet the physiological demands of the mother and fetus, with the conversion of 25(OH)D to 1.25-dihydroxyvitamin D [1.25(OH)_2_D] increasing approximately 2-fold throughout pregnancy (2). The fetus relies solely on an adequate maternal vitamin D intake to meet these demands (3). Current dietary reference values (DRVs) do not have a separate reference intake in relation to vitamin D intake for pregnant or lactating women (4, 5), and there is a lack of scientific consensus on the amount of vitamin D intake that is deemed optimal in populations of pregnant women, regardless of additional risk factors that contribute to low vitamin D status, such as obesity. Current UK DRVs for vitamin D in pregnant and nonpregnant women assume that an intake of 10 µg/d will achieve a target value for 25(OH)D concentrations of ≥25 nmol/L (5). The level of maternal adequacy must account for fetal and neonatal demands, although owing to a lack of trial data, it is also acknowledged that target thresholds for neonatal requirements are yet to be defined. It has been suggested that circulating 25(OH)D in newborns should be maintained above a minimum of 25–30 nmol/L (6).

Although limited in number, observational studies have shown that pregnant women with obesity [BMI (in kg/m^2^) >30) have a significantly lower vitamin D status than nonobese pregnant women (1, 7, 8). Andersen et al. reported that a 5 kg/m^2^ increase in BMI was associated with a decrease in vitamin D status of 3.7 nmol/L in pregnant women (9). Karlsson et al. reported that despite having a higher dietary vitamin D intake and similar supplement use, pregnant women with obesity had lower vitamin D status in the first trimester than normal-weight pregnant women (7).

The effect of vitamin D supplementation on maternal and neonatal status has been examined in previous systematic reviews (10–13), and supplementation studies (2, 14, 15) have consistently reported that vitamin D supplementation in a single or continued dose during pregnancy significantly increased maternal vitamin D status. Information regarding the relationship between maternal body weight and vitamin D status remains scarce. We hypothesize that the current vitamin D dosage recommendation for pregnant women of 10 μg/d may not be adequate in achieving levels of vitamin D sufficiency, particularly in those with a higher BMI in early pregnancy.

In this 2-arm parallel double-blind randomized trial, we compared the effects of 10 μg vitamin D_3_/d with those of 20 μg vitamin D_3_/d on maternal vitamin D status throughout pregnancy and in umbilical cord blood. We also evaluated within each supplementation group the influence of BMI on maternal vitamin D status throughout pregnancy and in umbilical cord blood.

## Methods

### Study design

**FIGURE 1:**
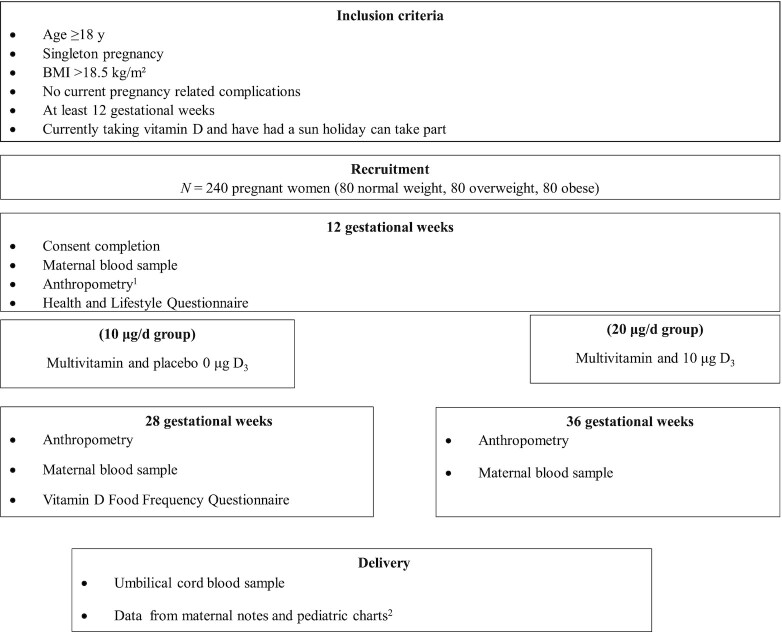
Flow diagram of study design. ^1^Anthropometric measurements included height (using a stadiometer) and weight in kg (using a TANITA, MC-780MA scale). ^2^Infant anthropometric measures at birth (weight, length, head circumference) and other measures relevant to the health status of the mother and child.

A 2-arm parallel double-blind randomized vitamin D intervention study of the association between maternal body weight and vitamin D status (MO-VITD study) was conducted and is being reported according to CONSORT guidelines (16). In total, 240 pregnant women received either 10 μg/d or 20 μg/d vitamin D_3_ from 12 gestational wk (GW) through to delivery (**Figure 1**).

### Participants

Pregnant women (*n* = 240, with equal numbers of normal weight, overweight, and obese) were recruited to the study during their first antenatal visit. The primary recruitment center was Altnagelvin Area Hospital, Western Health and Social Care Trust (WHSCT), in Northern Ireland. At the booking appointment (∼9–10 GW), all pregnant women in the WHSCT area received an information sheet from their healthcare provider with a detailed study outline. At the hospital clinic when women attended for their 12 GW antenatal scan, the researcher was present to answer any further questions and to take written informed consent from eligible participants.

The inclusion criteria were as follows: pregnant women ≥12 GW, aged ≥18 y, BMI ≥18.5, without current pregnancy-related complications, and having a singleton pregnancy. Exclusion criteria included multiple pregnancy, involvement in another research study, history of gastrointestinal, hepatic, renal, vascular, or hematological disorders. In addition, participants who had in vitro fertilization treatment, a history of neural tube–defect pregnancies, or active thyroid disease were excluded. Those participants who were taking a vitamin D containing supplement prior to the study were asked to only take the study supplements after enrollment. All participants provided written informed consent according to the Declaration of Helsinki. The study was reviewed by the Biomedical Sciences Research Ethics Filter Committee, Ulster University, 15/0041, and approved by the Office for Research Ethics Committees, Northern Ireland 15/NI/0068, and by the WHSCT (WT 14/49). The study was registered at ClinicalTrials.gov with the ID NCT02713009.

### Sample size

A sample size of 94 in each treatment group was calculated to detect a 10-nmol/L difference in 25(OH)D concentrations and provide 90% power at a significance level of *P* < 0.005 based on a previous study by Hollis et al. (2). To account for potential dropouts (those who discontinued the study, suffered miscarriage or pregnancy complications, or complied poorly with the study protocol) the sample size was increased by 25% to give a total of 120 in each treatment group.

### Randomization

Using MINIM randomization software (https://www-users.york.ac.uk/∼mb55/guide/minim.htm), a senior researcher not associated with the study randomly assigned participants to receive either 10 μg or 20 μg vitamin D_3_/d from 12 GW through to delivery. The randomization was stratified by BMI category. In the 10 µg/d group, 119 participants were given 2 tablets, 1 multivitamin tablet containing 10-μg vitamin D and a 0-μg vitamin D (placebo) tablet, and in the 20-µg/d group, 121 participants were given 2 tablets, 1 multivitamin tablet containing 10-μg vitamin D and a 10-μg vitamin D tablet. Participants were allocated an identification number in accordance with the random assignment. The participants and research investigators were blinded to the treatment allocation throughout the study period. The 10-μg/d dose in this intervention study was based on the current UK recommendation for pregnant women (5). The 20-μg/d dose was based on a previous study by Holmes et al. (17), in which the vitamin D status of non–supplement using pregnant women with obesity was 25 nmol/L; in order to raise this status to sufficiency (>50 nmol/L), it was estimated that 20 μg/d was required, as ∼1 μg of vitamin D may increase status by 1.2 nmol/L (18).

### Intervention

The multivitamins (Pregnacare,^®^ Vitabiotics) were supplied from Vitabiotics Ltd., and vitamin D_3_ and placebo were from Sona^®^ Nutrition. The placebo and vitamin D tablets were identical in size, color, and texture. The tablets were provided to the participants in 2 batches comprising weekly pill boxes, batch 1 from 12 to 28 GW and batch 2 from 28 to 40 GW. Participants were contacted by the researcher twice between 12 and 28 GW and again between 28 and 34 GW to check compliance. Following the 28-GW antenatal clinic visit, participants returned the pill boxes; all unused tablets were counted and recorded. This procedure was repeated at the 36 GW visit, and following this visit any remaining pill boxes were returned via a free post envelope after the birth. Adequate compliance was defined as supplement consumption >75%.

### Data collection

#### First antenatal visit

##### Anthropometry and health and lifestyle information

At the first antenatal visit at 12 GW, anthropometric measurements were taken, including height (using a stadiometer to the nearest 0.1 cm) and weight (to the nearest 0.1 kg using a TANITA, MC-780MA scale). BMI was categorized as normal weight at 18.5–24.9, overweight at 25.0–29.9, and obesity at >30.0. All measurements were carried out by trained researchers in a private environment within the clinic setting.

All participants completed a Health and Lifestyle Questionnaire at the 12 GW visit, which recorded information on age, social demographics, medication, supplementation use, and sun exposure, including sun bed/bathing use and sun holiday within the previous 6 mo. In addition, details from maternal notes were recorded, including GW, parity, blood pressure, smoking, and previous miscarriage.

##### Subsequent visits

Participants attended follow-up appointments at 28 and 36 GW. Anthropometric measurements were repeated as per the 12 GW visit. Information from maternal notes was again recorded, and at this timepoint also included information from the growth chart for the fetus and routine blood and urine sample results. Infant anthropometric measures at birth (weight, length, head circumference) and other measures relevant to the health status of the mother and child were recorded from maternal notes and pediatric charts after delivery of the infant.

##### Dietary intake

At the 28 GW visit, all participants completed a validated FFQ to assess vitamin D intake from foods (19).

##### Blood sample analysis

Nonfasting blood samples were collected at 12, 28, and 36 GW by a phlebotomist and umbilical cord blood samples were collected at delivery by the midwife on duty. A total of 20 mL (2 × 8–mL serum tubes and 1 × 4–mL plasma tube) of blood was collected, kept chilled, and processed within 3 h of collection. Plasma and serum aliquots were stored at −80°C until batch analysis. Stored serum samples were used for vitamin D analysis. Currently, the recognized marker of vitamin D status is the concentration of circulating 25(OH)D. Total serum 25(OH)D concentrations [25(OH)D_2_ plus 25(OH)D_3_] were measured by LC-tandem MS (LC-MS/MS) using a commercially available kit (API4000; AB SCIEX; Chromsystems Instruments and Chemicals GmbH; MassChrom 25-OH-Vitamin D_3_/D_2_). Vitamin D analysis was conducted at the biochemistry department of St James Hospital Dublin. This laboratory is fully accredited to ISO 15.189 Standard. Assay quality was monitored using in-house and third-party quality controls. Accuracy was determined using the National Institute of Standards and Technology vitamin D standard reference material 972. The laboratory also participates in the Vitamin D External Quality Assessment Scheme. Vitamin D status was classified into categories of sufficiency according to SACN guidelines and defined as deficient [25(OH)D <25 nmol/L], insufficient [25(OH)D 25–50 nmol/L], or sufficient [25(OH)D ≥50 nmol/L) (5). As safety measures, plasma intact parathyroid hormone (iPTH), serum calcium, and serum albumin were measured. iPTH concentrations were measured using a commercially available ELISA (Roche Elecsys PTH assay, Roche Diagnostics Ltd). Serum concentrations of calcium and albumin were measured using an automated clinical chemistry system (Ilab 650 Clinical Chemistry System, WERFEN). Albumin-adjusted calcium was calculated using the following formulas: adjusted calcium = measured total calcium + 0.02(40-albumin), and for albumin >45g/L: adjusted calcium = measured total calcium − 0.02(45-albumin).

### Statistical analysis

The statistical analyses were performed using SPSS (Statistical Package for the Social Sciences software, version 22; IBM). Data were assessed for normality using the Kolmogorov-Smirnov test. Data were presented as mean ± SD. Intention to treat analysis was conducted and multiple imputation was used to account for missing data. To aid the most reliable simulation of the monotonic imputation, all available baseline data (as presented in **Table 1**), including the assigned treatment group variable, were used to inform the imputation. Observed minimum and maximum acceptable values for variables were added where appropriate. Five imputation data sets and a combined pooled set were generated; the pooled imputation data are presented. The primary outcome changes in 25(OH)D status were: baseline (12 GW), midpoint (28 GW), and end of pregnancy (36 GW); serum vitamin D status (12, 28, and 36 GW); and cord serum vitamin D status at delivery. A general linear model, using repeated measures for 28 and 36 GW, adjusted for 25(OH)D at baseline, and using treatment group as a between-subject factor, was used to compare the effect of 10 μg/d and 20 μg/d supplementation on 25(OH)D concentrations. Independent sample *t*-tests were used to assess differences in maternal characteristics between treatment groups. Chi-square tests were used to determine differences in insufficiency/sufficiency and associations with other categorical variables, including smoking, education level, marital status, parity, medication use, dietary supplement use, recent sun holiday, sunbed/bathing use, and season. Bivariate correlations were performed between 25(OH)D concentrations and age, body weight, biochemical measures, and blood pressure. Differences in infant characteristics at birth between the 10-μg/d and 20-μg/d treatment groups were assessed using a general linear model, adjusted for baseline 25(OH)D concentrations. Results were considered significant when *P* < 0.05 in all analyses.

**TABLE 1:** Maternal characteristics at baseline in the 10-µg/d and 20-µg/d vitamin D treatment groups^1^

	10 µg/d (*n* = 118)	20 µg/d (*n* = 121)
Age, y	29.7 ± 5.1	29.5 ± 5.5
Weight, kg	74.7 ± 15.9	74.4 ± 15.9
Height, m	1.63 ± 0.06	1.63 ± 0.06
BMI, kg/m^2^	28.1 ± 5.7	27.8 ± 5.4
Normal weight	39 (33.1)	41 (33.9)
Overweight	39 (33.1)	40 (33.1)
Obese	40 (33.9)	40 (33.1)
Gestational wk	13.0 ± 1.4	12.8 ± 1.4
Blood pressure, mm Hg		
Systolic	119.3 ± 11.1	120.5 ± 11.6
Diastolic	71.8 ± 8.5	72.7 ± 9.0
Smoker	16 (13.5)	14 (11.5)
Parity		
0	53 (45.0)	43 (35.5)
1	34 (28.8)	48 (39.7)
2^+^	30 (25.4)	28 (23.1)
Previous miscarriage	41 (34.7)	30 (24.7)
Education level		
Secondary	38 (32.2)	36 (29.7)
Diploma	18 (15.3)	23 (19.0)
Degree	38 (33.9)	38 (31.4)
Postgraduate	16 (13.5)	14 (12.0)
Other	6 (5.0)	6 (5.0)
Marital status		
Married	60 (50.8)	62 (51.2)
Unmarried	58 (49.2)	59 (48.8)
Regular medication	15 (12.7)	8 (6.6)
Medical illnesses	13 (11.0)	9 (7.4)
Vitamin D supplement use		
User	66 (55.9)	81 (66.9)
Nonuser	52 (44.1)	40 (33.1)
Sun holiday past 6 mo	20 (16.9)	23 (19.0)
Sunbathing/sunbed last mo	7 (6.0)	11 (9.1)
Season at enrollment		
Winter (October–March)	61 (51.7)	67 (55.4)
Summer (April–September)	57 (48.3)	54 (44.6)

^1^Data are presented as means ± SDs or *n* (%). Differences between 10-µg/d and 20-µg/d groups were assessed by independent sample *t*-test or chi-square test as appropriate, *P* < 0.05 considered significant. No significant differences between any parameters at baseline.

## Results

### Recruitment

**FIGURE 2:**
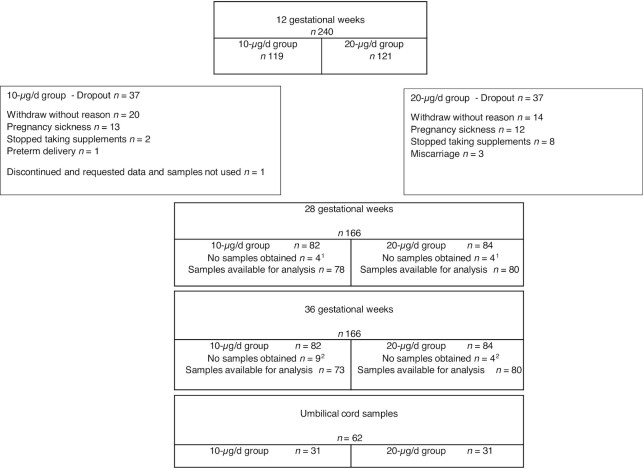
Flow diagram of study participants. ^1^Reason for no samples obtained: pregnancy sickness (*n* = 2 in 10-µg/d group, *n* = 2 in 20-µg/d group), unable to attend appointment (*n* = 2 in 10-µg/d group,*n* = 2 in 20-µg/d group). ^2^Reasons for no samples obtained: pregnancy sickness (*n* = 1 in 20-g/d group), unable to attend appointment (*n* = 5 in 10-µg/d group, *n* = 2 in 20-µg/d group), preterm delivery (*n* = 4 in 10-µg/d group, *n* = 1 in 20-µg/d group).

A total of 240 pregnant women completed the 12-GW baseline visit (119 in the 10-µg/d group and 121 in the 20-µg/d group); samples were available for all but 1 participant from the 10-µg/d group who withdrew from the study. A total of 74 participants withdrew from the study after baseline (37 in the 10-µg/d group and 37 in the 20-µg/d group), and there was no difference in dropout rates according to BMI category. A total of 166 participants completed the 28-GW visit, of which 158 provided blood samples (78 in the 10-µg/d group and 80 in the 20-µg/d group). At the 36-GW visit, a total of 166 participants completed the study measurements, and of these, 153 participants provided blood samples (73 in the 10-µg/d group and 80 in the 20-µg/d group); details of the reasons for withdrawal are explained in **Figure 2**. Compliance with the intervention was high, with no difference between the 10-µg/d and 20-µg/d treatment groups: 92.4% compared with 91.7%, respectively. There were no adverse events reported by participants on this study.

### Maternal characteristics at baseline

A total of 239 pregnant women were included in this analysis, 118 in the 10-µg/d group and 121 in the 20-µg/d group. There were no significant differences in any maternal characteristics between treatment groups at baseline (Table 1).

### Maternal vitamin D status

Mean maternal 25(OH)D concentrations at each timepoint are shown in **Table 2**. Maternal 25(OH)D concentrations increased from baseline to the 36-GW timepoint in both the 10-µg/d and 20-µg/d groups, with a higher increase observed in the 20-µg/d group (*P* < 0.001).

**TABLE 2:** Maternal 25(OH)D concentrations by BMI category throughout pregnancy and in umbilical cord at birth in each treatment group^1^

	Baseline			28 gestational wk			36 gestational wk			Umbilical cord		
	10 µg/d (*n* = 118)	20 µg/d (*n* = 121	*P* value	10 µg/d (*n* = 118)	20 µg/d (*n* = 121	*P* value	10 µg/d (*n* = 118)	20 µg/d (*n* = 121	*P* value	10 µg/d (*n* = 31	20 µg/d (*n* = 31	*P* value
25(OH)D, nmol/L												
All	52.2 ± 22.9	52.0 ± 20.5	0.920	69.5 ± 25.6	82.8 ± 30.7	<0.001	80.8 ± 28.2	94.4 ± 33.2	<0.001	33.4 ± 12.8	36.4 ± 14.7	<0.001
Normal weight (*n* = 80)	55.9 ± 26.3	59.2 ± 19.8	0.516	71.4 ± 23.8	91.9 ± 27.8	<0.001	85.7 ± 25.0	106.7 ± 31.1	<0.001	35.6 ± 12.6	43.0 ± 14.3	<0.001
Overweight (*n* = 79)	51.0 ± 22.2	48.7 ± 18.7	0.620	72.9 ± 25.8	81.6 ± 29.6	<0.001	81.9 ± 24.9	93.9 ± 31.3	<0.001	33.8 ± 11.0	35.4 ± 14.0	0.005
Obese (*n* = 80)	49.7 ± 19.7	48.0 ± 21.2	0.716	64.3 ± 27.0	74.6 ± 32.2	0.092	74.8 ± 30.6	82.1 ± 33.4	0.480	30.7 ± 14.1	30.7 ± 12.8	0.855

1 Data are means ± SD. *P* differences in 25(OH)D between 10-µg/d and 20-µ/d treatment groups were assessed using a general linear model, adjusted for baseline 25(OH)D at 28 and 36 gestational wk and umbilical cord. Data at 28 and 36 gestational wk include imputed data; blood samples available for analysis at 28 gestational wk were *n* = 78 and *n* = 80, and at 36 gestational wk were *n* = 73 and *n* = 80, in the 10-µg/d and 20-µg/d treatment groups, respectively.

Maternal 25(OH)D concentrations at the baseline visit were not significantly different between the 10-µg/d and 20-µg/d groups; 52.2 ± 22.9 compared with 52.0 ± 20.5 nmol/L, *P* = 0.920. At the 28-GW and 36-GW timepoints, 25(OH)D concentrations were significantly lower in the 10-µg/d group than the 20-µg/d group (28 GW: 69.5 ± 25.6 compared with 82.8 ± 30.7 nmol/L, *P* < 0.001; 36 GW: 80.8 ± 28.2 compared with 94.4 ± 33.2 nmol/L, *P* < 0.001).

Maternal 25(OH)D concentrations at baseline were not significantly different between the 10-µg/d and 20-µg/d groups in any of the BMI categories. At the 28- and 36-GW timepoints, in normal weight and overweight women, 25(OH)D concentrations were significantly higher in the 20-µg/d group than the 10-µg/d group. In women with obesity, this trend for a higher 25(OH)D concentration in the 20-µg/d group compared with the 10-µg/d group was evident at 28 GW but not at 36 GW, albeit these differences were not statistically significant.

### Infant characteristics

A total of 164 infants (81 in the 10-µg/d group and 83 in the 20-µg/d group) were born to mothers who completed the intervention (**Table 3**). At delivery there were no significant differences in gestational age or mode of delivery between the 10-µg/d and 20-µg/d treatment groups. Nor were there any differences between birth weight, length, Apgar scores, or sex in infants born in each treatment group. Significantly more infants categorized as normal weight were born to mothers in the 10-µg/d treatment group, whereas there was a higher percentage of infants with macrosomia born to mothers in the 20-µg/d treatment group (*P* = 0.026). Infants born to mothers in the 10-µg/d group had a significantly lower head circumference than infants born to mothers in the 20-µg/d group (34.7 ± 2.4 compared with 35.8 ± 2.7 cm, *P* = 0.003).

**TABLE 3:** Infant characteristics in each vitamin D treatment group^1^

	10 µg/d (*n* = 81)	20 µg/d (*n* = 83)	*P* value
Gestation at delivery, wk	39.4 ± 4.8	39.8 ± 2.8	0.453
Mode of delivery			
Vaginal delivery	41 (50.6)	38 (45.8)	0.561
Caesarean section	33 (40.7)	36 (43.4)	
Forceps	6 (7.4)	5 (6.0)	
Vacuum	1 (1.2)	4 (4.8)	
Apgar^2^			
1 min	8.4 ± 1.7	8.5 ± 1.3	0.548
5 min	8.9 ± 1.3	9.0 ± 0.7	0.451
Anthropometry			
Birth weight, g	3429.1 ± 829.4	3638.9 ± 639.9	0.095
Low birth weight	3 (3.7)	3 (3.6)	0.026
Normal birth weight	67 (82.7)	63 (75.9)	
Macrosomia	11 (13.6)	17 (20.5)	
Birth length, cm	52.2 ± 4.9	52.9 ± 4.0	0.397
Head circumference, cm	34.7 ± 2.4	35.8 ± 2.7	0.003
Sex			
Male	37 (45.7)	43 (51.8)	0.439
Female	44 (54.3)	40 (48.2)	

1 Data are presented as means ± SDs or *n* (%).

2 Apgar test performed at 1 and 5 min after birth to assess infant health. Scores ≥ 7 considered normal, 4–6 low, and ≤ 3 regarded as critically low. Birth weight defined by WHO: low birth weight <2500 g and macrosomia >4000 g. *P*, differences between 10-µg/d and 20-µg/d groups assessed using a general linear model, adjusted for 25(OH)D at baseline, or chi-square test as appropriate, *P* < 0.05 considered significant.

### Umbilical cord vitamin D status

Sixty-two umbilical cord blood samples were collected (31 samples in each of the 10-µg/d and 20-µg/d groups) (Table 2). Umbilical cord blood 25(OH)D concentrations were significantly lower in infants born to mothers in the 10-µg/d compared with the 20-µg/d group (33.4 ± 12.8 compared with 36.4 ± 14.7 nmol/L, *P* < 0.001). When examined by BMI category, umbilical cord 25(OH) concentrations were significantly lower in the 10-µg/d group than the 20-µg/d group in both normal weight and overweight women (normal weight 35.6 ± 12.6 compared with 43.3 ± 14.3 nmol/L, *P* < 0.001; overweight 33.8 ± 11.0 compared with 35.4±14.0 nmol/L, *P* = 0.005). No differences were observed between the 10-µg/d compared with 20-µg/d groups in the umbilical cord 25(OH)D concentrations in infants born to mothers with obesity (30.7±14.1 compared with 30.7 ± 12.8 nmol/L, *P* = 0.855). Umbilical cord 25(OH)D concentrations were positively correlated with maternal 25(OH)D concentrations at baseline, 28, and 36 GW (*r* = 0.552, *P* < 0.0001; *r* = 0.585, *P* < 0.0001; *r* = 0.784, *P* < 0.0001) respectively. When examined by categories of insufficiency (<50 nmol/L) and sufficiency (≥50 nmol/L), on average, 45% of participants had an insufficient status on entering pregnancy (41.5% compared with 48.8% in the 10-µg/d and 20-µg/d groups respectively) (**Table 4**). In participants who reported vitamin D supplement use prior to commencing our study, 40.8% and 57.6% (10-µg/d and 20-µg/d groups respectively) were still classified as insufficient at 12 GW (data not shown).

**TABLE 4:** Prevalence of maternal 25(OH)D insufficiency and sufficiency throughout pregnancy and in umbilical cord at birth, in each treatment group^1^

	Baseline		28 gestational wk		36 gestational wk		Umbilical cord	
	10 µg/d	20 µg/d	10 µg/d	20 µg/d	10 µg/d	20 µg/d	10 µg/d	20 µg/d
All	n 118	n 121	n 118	n 121	n 118	n 121	n 31	n 31
<50 nmol/L	41.5	48.8	21.2	14.8	12.7	9.9	77.4	54.8
≥50 nmol/L	58.5	51.2	78.8	85.2	87.3	90.1	22.6	45.2
Normal weight								
<50 nmol/L	35.9	34.1	20.5	9.8	10.3	4.9	81.8	38.5^2^
≥50 nmol/L	64.1	65.9	79.5	90.2	89.7	95.1	18.2	62.5
Overweight								
<50 nmol/L	43.6	55.0	15.4	12.5	7.7	7.5	88.9	60.0
≥50 nmol/L	56.4	45.0	84.6	87.5	92.3	92.5	11.1	40.0
Obese								
<50 nmol/L	45.0	57.5	27.5	22.5	20.0	17.5	63.6	75.0
≥50 nmol/L	55.0	42.5	81.5	77.5	80.0	82.5	36.4	25.0

1 Data are percentages, with differences in maternal and infant classifications of insufficiency (<50 nmol/L) and sufficiency (≥50 nmol/L) between treatment groups assessed using a chi-square test. Data at 28 and 36 gestational wk include imputed data; blood samples available for analysis at 28 gestational wk were *n* = 78 and *n* = 80, and at 36 gestational wk were *n* = 73 and *n* = 80, in the 10-µg/d and 20-µg/d treatment groups, respectively.

2 Significant difference in percentage prevalence of insufficiency and sufficiency in umbilical cord blood of normal weight women, *P =* 0.028. *P* < 0.05 considered significant.

**FIGURE 3:**
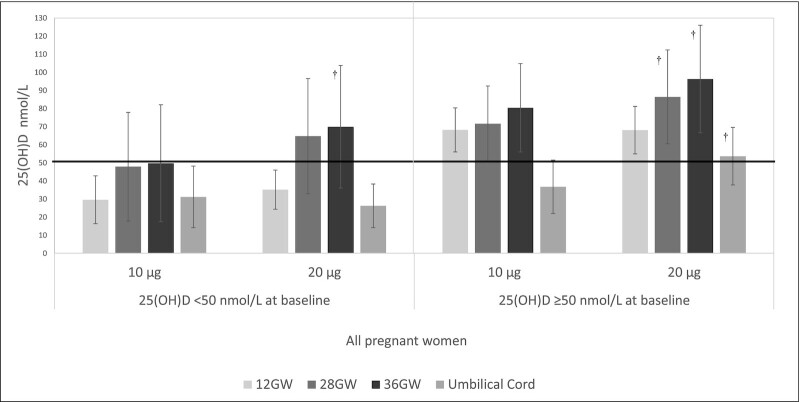
25(OH)D concentrations according to categorization of insufficiency or sufficiency at baseline and corresponding response to treatment. 25(OH)D concentrations in 10-µg/d and 20-µg/d treatment groups in all pregnant women at each timepoint and in umbilical cord, for those who started pregnancy with 25(OH)D concentrations <50 nmol/L (*n* = 108) and ≥50 nmol/L (*n* = 131). Line at 50 nmol/L indicates sufficiency. GW, gestational weeks. †Indicates significant difference (*P* < 0.05) between 10-µg/d and 20-µg/d treatment groups at the corresponding timepoint, assessed by independent *t*-test. Time by treatment interaction *P* < 0.001.

**Figure 3** presents the 25(OH)D response to 10 µg/d and 20 µg/d supplementation at each timepoint, examined separately by those who had a baseline 25(OH)D concentration <50 nmol/L compared with ≥50 nmol/L. In those who were categorized as insufficient at baseline, mean 25(OH)D concentrations remained within the insufficiency category throughout pregnancy in the 10-µg/d group. However, in the 20-µg/d group, those categorized as insufficient at baseline reached mean 25(OH)D concentrations within the sufficiency category at 28 and 36 GW. Pregnant women who started pregnancy with a sufficient 25(OH)D concentration maintained mean 25(OH)D status within the sufficiency range throughout pregnancy in both the 10-µg/d and 20-µg/d groups. When examined by BMI category, similar findings were obtained in the normal weight, overweight, and obese categories.

In those who started pregnancy with an insufficient status, corresponding mean umbilical cord 25(OH)D concentrations were found to be within the insufficiency range in infants born to mothers in both the 10-µg/d and 20-µg/d groups (31.2 ± 17.0 compared with 26.3 ± 12.0 nmol/L, *P* = 0.461) respectively, whereas when the mother had a sufficient status at baseline, mean umbilical cord blood 25(OH)D concentrations were within the sufficiency category in infants born to mothers in the 20-µg/d group but not in infants born to mothers in the 10-µg/d group (53.7 ± 15.9 compared with 36.8 ± 14.7 nmol/L, *P* = 0.001) respectively.

Similar trends which were dependent on maternal insufficiency/sufficiency at baseline were observed in the umbilical cord blood 25(OH)D concentrations of infants born to normal weight and overweight pregnant women. However, in women with obesity who started pregnancy with an insufficient status, mean umbilical cord blood status was found to be classified as deficient regardless of 10 µg/d compared with 20 µg/d treatment (19.4 ± 20.2 compared with 19.5 ± 9.4 nmol/L, *P* = 0.992). In contrast, women with obesity who started pregnancy with a sufficient status had mean umbilical cord blood concentrations above deficiency and within the insufficiency range in both the 10-µg/d compared with 20-µg/d treatment groups (40.2 ± 18.4 compared with 44.2 ± 15.6, *P* = 0.747). When 36 GW maternal 25(OH)D concentrations were related to umbilical cord blood 25(OH)D concentrations, women who had an insufficient status in late pregnancy were found to have umbilical cord blood levels classified as deficient, regardless of 10 µg/d compared with 20 µg/d treatment (12.7 ± 2.6 compared with 23.2 ± 16.7 nmol/L, *P* = 0.460). In contrast, when 36 GW maternal 25(OH)D concentrations were sufficient, corresponding umbilical cord blood 25(OH)D concentrations were classified as insufficient (and out of the deficiency category) in both the 10-µg/d compared with 20-µg/d treatment groups (37.1 ± 14.3 compared with 44.3 ± 19.2 nmol/L, *P* = 0.118).

There was no difference in the calculated percentage placental transfer of maternal 25(OH)D concentrations to the umbilical cord blood 25(OH)D concentration between the 10-µg/d and 20-µg/d groups (41% compared with 42% respectively); nor were there any differences in placental transfer of 25(OH)D between the 10-µg/d and 20-µg/d groups among pregnant women of normal weight, overweight, and obesity.

Pregnant women with obesity in the 10-µg/d group who had an insufficient 25(OH)D concentration (<50 nmol/L) at baseline transferred a lower 25(OH)D concentration to their infants compared with women who received 20 µg/d vitamin D (21.5% compared with 32.8%, *P* = 0.046).

There were no differences in maternal adjusted calcium or PTH concentrations at any timepoint between the 10-µg/d and 20-µg/d treatment groups [adjusted calcium (mmol/L): baseline 2.29 ± 0.10 compared with 2.30 ± 0.11; 28 GW 2.27 ± 0.11 compared with 2.29 ± 0.08; 36 GW 2.27 ± 0.11 compared with 2.28 ± 0.10; PTH (pg/mL): baseline 21.2 ± 7.8 compared with 21.9 ± 9.0; 28 GW 23.4 ± 9.1 compared with 22.8 ± 8.2; 36 GW 23.8 ± 9.3 compared with 23.5 ± 9.0, 10-µg/d and 20-µg/d groups respectively]. There were no associations found between maternal adjusted calcium concentrations and maternal 25(OH)D concentrations in any timepoint. At 12 and 36 GW maternal PTH concentrations were negatively correlated with maternal 25(OH)D concentrations (*r* = −0.343, *P* < 0.0001; *r* = −0.130, *P* = 0.045). Maternal 25(OH)D concentrations at 36 GW were positively correlated with infant birth weight and head circumference (*r* = 0.157, *P* = 0.45; *r* = 0.187, *P* = 0.017) respectively.

## Discussion

Despite global recommendations for vitamin D supplementation for the maintenance of maternal and fetal 25(OH)D concentrations, the adequacy of current guidelines alongside the influence of maternal obesity in response to vitamin D supplementation in pregnancy has not been adequately addressed. When we assessed 25(OH)D concentrations at the total group level of 239 pregnant women, both 10 µg/d and 20 µg/d of vitamin D_3_ increased average 25(OH)D to a sufficient status (≥50 nmol/L) for the duration of pregnancy. The increase in 25(OH)D was higher in the 20-µg/d group compared with the 10-µg/d group and this finding was also reflected in umbilical cord concentrations. We have shown that normal weight and overweight pregnant women in the 20-µg/d group had significantly higher maternal and umbilical cord 25(OH)D compared with normal weight and overweight pregnant women in the 10-µg/d group, with no differences in 25(OH)D between treatment groups in women with obesity. This shows that pregnant women of normal weight and overweight had a higher response to the higher vitamin D dose (20 µg/d) compared with pregnant women with obesity. This observed difference may be related to sequestration of vitamin D in adipose tissue in those with obesity. Other maternal physiological changes including gestational weight gain, hemodilution, and volumetric dilution are also postulated to influence vitamin D status (20). Hemodilution and gestational weight gain are thought to have a similar effect on vitamin D status across different BMI categories (7), however a dearth of knowledge remains on this issue.

Although previous dose response studies in pregnant women have demonstrated increases in maternal 25(OH)D concentrations (21–23), the definition of adequate or optimal 25(OH)D concentrations for maternal health remains controversial, and the appropriateness of comparing concentrations in pregnancy with target thresholds established for nonpregnant adults is debated. Similarly, there are no known thresholds for 25(OH)D concentrations in umbilical cord indicative of improved infant health outcomes. In addition, there is no global consensus as to what constitutes an adequate vitamin D supplementation level during pregnancy. The current UK recommendation of 10 µg vitamin D/d for all pregnant and lactating women has been set with the aim of preventing maternal and infant deficiency (>25 nmol/L) (5). The European Food Safety Authority (EFSA) has noted that currently, data are not available to suggest a different target value for 25(OH)D concentration for pregnant women compared with nonpregnant women (4), and the Institute of Medicine suggest a target threshold of >30 nmol/L (24).

We have shown that in women who commenced pregnancy with an insufficient status, mean 25(OH)D concentrations did not reach sufficiency at any stage in pregnancy in the 10-µg/d group, highlighting that the current UK SACN recommendation (5) may be inadequate to achieve a sufficient status in those women who start pregnancy with a low 25(OH)D. In our study, 45% of all participants had an insufficient status upon entering pregnancy. In participants who reported vitamin D supplement use prior to commencing our study, 40.8% and 57.6% (10-µg/d and 20-µg/d groups respectively) were still classified as insufficient at 12 GW, despite adherence to current public health vitamin D supplementation guidelines. A study by Holmes et al. in 2009 reported that over 90% of pregnant women living in the same latitude as the current study had insufficient 25(OH)D, even with supplement use (17); our study shows that low 25(OH)D in pregnancy, even with vitamin D supplement use as per the recommended guideline, remains a major public health concern. Given the large population of pregnant women, recruited across all seasons, our findings can be considered reflective of the current 25(OH)D concentration of women in early pregnancy, who, even when adherent to the current recommendation of 10 µg/d, may not reach 25(OH)D sufficiency at any stage of pregnancy. In contrast, in those who started pregnancy with sufficient status, mean 25(OH)D concentrations remained sufficient throughout pregnancy in both the 10-µg/d and 20-µg/d groups. Whereas these findings may be viewed as target achieving for the UK SACN (5) and Institute of Medicine (24) guidelines to prevent maternal deficiency (<25–30 nmol/L), 45% of women in this cohort did not reach maternal sufficiency despite taking the recommended 10 µg/d vitamin D throughout pregnancy. In addition, even when starting pregnancy with a sufficient status, the 10 µg/d UK SACN supplementation strategy may be inadequate to achieve a sufficient 25(OH)D umbilical cord concentration. Findings in normal weight and overweight pregnant women showed that 10 µg vitamin D_3_/d is enough to prevent pregnant women and their infant from vitamin D deficiency; however, it is not enough to ensure maternal or umbilical cord sufficiency. Our findings are particularly important in the context of maternal obesity, with the UK now having the highest level of maternal obesity in Europe (25). Pregnant women with obesity who started pregnancy with an insufficient 25(OH)D had deficient umbilical cord concentrations in both the 10-µg/d and 20-µg/d groups, despite mothers having sufficient 25(OH)D at 36 GW; this was not observed in mothers who were either normal weight or overweight. This result supports previous findings that neonates of mothers with obesity have significantly lower umbilical cord 25(OH)D compared with neonates of normal weight mothers (26, 27) despite no differences in maternal 25(OH)D (28). This result again supports our finding that the current recommendation of 10 µg/d vitamin D during pregnancy may be inadequate particularly based on the high prevalence of vitamin D insufficiency and on the current high prevalence of maternal obesity. Our findings show that current strategies may potentially put infants born to mothers with obesity at high risk for vitamin D deficiency and poor in-utero bone development (29, 30).

The vitamin D status of the neonate has been shown to be highly correlated with maternal 25(OH)D concentrations (31–33) accounting for 60–80% of infants' status at delivery (34, 35). The current study found umbilical cord 25(OH)D much lower than previously reported, at 42% of maternal 25(OH)D in late pregnancy, with no difference between the 10-µg/d compared with 20-µg/d treatment groups. We report that the calculated percentage of placental transfer was significantly lower among pregnant women with obesity who started pregnancy with insufficient 25(OH)D and received 10 µg/d compared with pregnant women with obesity who started pregnancy with insufficient 25(OH)D and received 20 µg/d (21.5% and 32.8%). Given the importance of maternal 25(OH)D to the infant, our data suggest that a higher target vitamin D intake of ≥20 µg/d is necessary for improved maternal and infant vitamin D status. The strengths of this study include the measurement of 25(OH)D using the gold standard method of LC-MS/MS (24). As safety measures, maternal adjusted calcium and parathyroid hormone concentrations were measured and remained unchanged throughout pregnancy with no differences observed between treatment groups. Compliance with the intervention was estimated as >91% in both treatment groups. There were only 62 umbilical cord blood samples available for analysis, and thus the findings should be interpreted with caution. Future studies may consider the extent of the interaction between 25(OH)D status and BMI and based on our current findings, we now have preliminary data on which to base more precise power calculations.

In conclusion, whilst both 10 µg and 20 µg vitamin D/d can adequately raise 25(OH)D in pregnancy, we have demonstrated that the current UK recommendation of 10 µg vitamin D/d can achieve a sufficient status, but these levels of sufficiency are not achieved by those who start pregnancy with a low 25(OH)D status. In those who start pregnancy with a low 25(OH)D status, 20 µg vitamin D/d may be needed to achieve sufficiency. This risk is further increased in women with obesity who enter pregnancy with a low vitamin D status. Umbilical cord 25(OH)D concentrations were found to be deficient in women with obesity who started pregnancy with a low vitamin D status, potentially putting infants at high risk for poor in utero bone development. This research highlights the need for a revised maternal policy on maternal vitamin D supplementation guidelines, particularly considering our findings on maternal obesity and the implications for related infant umbilical cord 25(OH)D concentrations.

## Data Availability

Data described in the manuscript, code book, and analytic code will be made available upon request pending application and approval.

## Notes

PhD Consumables supported by the Royal Embassy of Saudi Arabia. Vitabiotics Ltd. and Sona nutrition provided the vitamin supplements. Only the named authors had input to study design, data interpretation, and study conclusions.

Abbreviations used: DRV, dietary reference value; GW, gestational weeks; iPTH, intact parathyroid hormone; LC-MS/MS, LC–tandem MS; WHSCT, Western Health and Social Care Trust; 25(OH)D, 25-hydroxyvitamin D concentrations.
